# Determinants of Sagittal Alignment Following First Metatarsophalangeal Arthrodesis: The Influence of Native Anatomy, Fixation Construct, and Surgeon Experience

**DOI:** 10.3390/jcm15218698

**Published:** 2026-11-07

**Authors:** Thomas Stumpner, Paul Pöchgraber, Dietmar Mattausch, Reinhold Ortmaier, Martin Bischofreiter, Harald Kindermann, Alexander Auffarth, Simon Recheis

**Affiliations:** 1 Department of Orthopaedic Surgery, Ordensklinikum Linz Barmherzige Schwestern Vinzenzgruppe Center of Orthopedic Excellence, Teaching Department of the Paracelsus Medical University Salzburg, Seilerstätte 4, 4020 Linz, Austria; 2 Department of Marketing and Electronic Business, University of Applied Sciences Upper Austria, Wehrgrabengasse 1, 4400 Steyr, Austria; 3 Department of Orthopedics and Traumatology, Paracelsus Medical University, Müllner Hauptstraße 48, 5020 Salzburg, Austria

**Keywords:** first metatarsophalangeal arthrodesis, hallux rigidus, sagittal alignment, dorsiflexion angle, nonunion, fixation construct, surgeon case volume

## Abstract

**Background/Objectives**: Optimal sagittal alignment after first metatarsophalangeal (MTP) joint arthrodesis is important for postoperative function, but its determinants remain poorly understood. This study evaluated the associations of fixation method, native sagittal anatomy, and surgeon case volume with postoperative alignment. **Methods**: A retrospective cohort study included 272 primary first MTP arthrodeses for end-stage hallux rigidus performed between 2012 and 2022. Fixation consisted of compression screws (*n* = 149) or a dorsal plate with a compression screw (*n* = 123). Sagittal alignment was assessed on weight-bearing radiographs using the dorsiflexion angle (DFA) and hallux ground angle (HGA); secondary parameters were the metatarsal declination angle (MDA) and hallux valgus angle (HVA). Surgeons were stratified by case volume as high (>30), medium (10–30), or low (<10 arthrodeses). Radiographic nonunion was assessed at 6 months. Group comparisons and correlation analyses evaluated associations between fixation construct, native anatomy, surgeon case volume, and postoperative alignment. **Results**: Plate fixation was associated with greater postoperative hallux dorsiflexion relative to both the first metatarsal (DFA: 25.4 degrees vs. 21.9 degrees, *p* < 0.001) and the ground plane (HGA: 6.4 degrees vs. 4.15 degrees, *p* < 0.001), whereas HVA was similar between groups (*p* = 0.545). The plate group demonstrated a greater MDA (21.3 degrees vs. 20.5 degrees, *p* = 0.028), and MDA correlated positively with DFA in both fixation groups (screw: r = 0.19, *p* = 0.042; plate: r = 0.29, *p* = 0.006). Surgeon case volume was associated with postoperative sagittal alignment, with lower-volume surgeons demonstrating greater HGA values (*p* = 0.002). The overall radiographic nonunion rate was 9.6%. **Conclusions**: Postoperative sagittal alignment was associated with native anatomy, fixation construct, and surgeon case volume. Hallux position should be individualized according to native anatomy and confirmed by functional intraoperative assessment rather than relying on implant geometry or predefined radiographic target angles alone. Level of Evidence: Level III, retrospective comparative study.

## 1. Introduction

First metatarsophalangeal (MTP I) joint arthrodesis represents the gold standard surgical treatment for advanced hallux rigidus [1,2]. Long-term follow-up studies of up to 31 years have demonstrated favorable functional outcomes and high patient satisfaction [3,4,5,6,7,8].

Postoperative function depends on both correct alignment and reliable osseous fusion of the arthrodesis [9,10]. Sagittal malalignment is a major cause of patient dissatisfaction and reoperation after MTP arthrodesis [11]. A dorsiflexion angle of approximately 20 to 30 degrees and a physiological valgus alignment of 5 to 25 degrees are commonly recommended [12]; however, these radiographic targets may not account for individual anatomical variation. In particular, differences in sagittal orientation of the first ray, reflected by the metatarsal declination angle, may influence the amount of hallux dorsiflexion required to achieve physiological rollover. Consequently, intraoperative positioning should be guided by functional assessment under simulated weight-bearing conditions [10]. The surgeon must balance sufficient dorsiflexion to allow physiological rollover during gait with adequate toe-tip contact for push-off [12,13]. Achieving this balance requires careful intraoperative assessment, as excessive dorsiflexion may lead to shoe conflict, prevent effective toe-off [12,14], and is associated with increased metatarsal pressure [15], whereas insufficient dorsiflexion results in increased pressure beneath the hallux [15,16] and may impair gait [14].

Successful osseous fusion requires precise joint preparation with complete cartilage removal, creation of congruent surfaces to achieve optimal bone-to-bone contact, and stable fixation [17]. During the study period, fixation strategies evolved from primary use of compression screws toward anatomically contoured locking plates combined with an interfragmentary compression screw. Combined lag-screw and plate constructs are considered the most biomechanically stable fixation constructs for MTP I arthrodesis [18,19]. Although contoured plates may facilitate reproducible fixation, their predefined geometry may influence the final arthrodesis position [14,20]. However, the extent to which fixation method affects postoperative alignment in clinical practice remains unclear.

Therefore, the aim of this study was to assess the association of fixation method, native sagittal anatomy, and surgeon case volume with postoperative sagittal alignment and radiographic nonunion following MTP I arthrodesis.

## 2. Materials and Methods

This retrospective single-center cohort study included all consecutive primary first metatarsophalangeal (MTP I) joint arthrodeses performed for end-stage hallux rigidus between January 2012 and December 2022. Revision arthrodeses and concomitant procedures affecting first-ray alignment were excluded. A total of 272 arthrodeses in 252 patients met the eligibility criteria and constituted the final study cohort.

### 2.1. Surgical Procedure and Postoperative Care

Arthrodesis was performed through a standard dorsal approach. Joint surfaces were prepared using a conical cup-and-cone reaming system (DePuy Synthes, Synthes GmbH, Oberdorf, Switzerland), followed by multiple drill perforations of the subchondral surfaces to promote bone healing. Hallux position was determined intraoperatively using simulated weight-bearing with a flat plate, aiming for neutral rotation and physiological valgus alignment. Sagittal alignment was adjusted to achieve slight dorsiflexion, with the toe tip positioned approximately 1–2 mm above the plate and the interphalangeal joint resting in slight flexion. The plate was then tilted to simulate rollover, ensuring sufficient dorsiflexion for physiological progression while avoiding hyperextension of the interphalangeal joint. Fixation was achieved using either two or three 4 mm partially threaded compression screws or a combination of an interfragmentary compression screw and a locking plate (Medartis AG, Basel, Switzerland), with plate fixation increasingly replacing screw fixation over the study period. Postoperative weight-bearing was determined by intraoperative stability and ranged from immediate full weight-bearing to 2–4 weeks of partial weight-bearing.

### 2.2. Radiological Assessment

**Figure 1:**
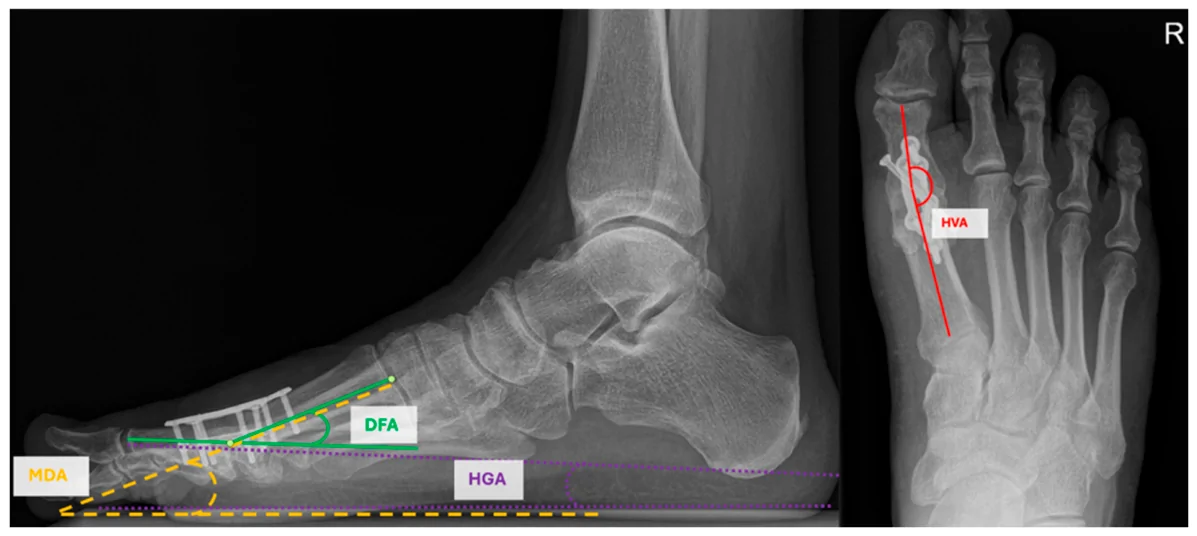
Radiographic assessment of alignment after MTP I arthrodesis showing HVA (dorsoplantar view) and DFA, MDA and HGA (lateral view).

Postoperative radiographic assessment was performed on standardized weight-bearing radiographs. The following parameters were measured (Figure 1):

- Metatarsal declination angle (MDA): angle between the longitudinal axes of the first metatarsal and the ground contact plane on lateral view;
- Hallux valgus angle (HVA): angle between the longitudinal axes of the first metatarsal and proximal phalanx on dorsoplantar view;
- Dorsiflexion angle (DFA): angle between the longitudinal axis of the first metatarsal and the proximal phalanx on lateral view;
- Hallux ground angle (HGA): angle between the longitudinal axis of the proximal phalanx of the hallux and the ground contact plane on lateral view.

Nonunion was defined as the absence of radiographic osseous fusion at six months postoperatively, as independently assessed by two observers. Surgeons were grouped according to individual case volume during the study period into high-volume (>30 procedures), medium-volume (10–30 procedures), and low-volume (<10 procedures) surgeons.

Missing radiographic measurements were not imputed. Analyses were performed using available cases for each radiographic parameter, and the number of observations included in each analysis is reported accordingly.

### 2.3. Statistical Analysis

Statistical analyses were conducted on an exploratory basis. The study cohort was characterized using descriptive summary statistics of demographic and baseline characteristics. Continuous baseline variables were compared between fixation groups using independent-samples *t* tests, whereas categorical variables were compared using chi-square tests. Differences in postoperative radiographic parameters between screw and plate fixation were assessed using the nonparametric Mann–Whitney U test because of the presence of outliers. Associations between metatarsal declination angle and postoperative dorsiflexion angle were evaluated using two-sided Pearson correlation coefficients. Additionally, medium- and low-volume surgeons were pooled and compared with the high-volume surgeon for radiographic parameters using Welch’s *t* test.

All statistical tests were two-sided, with the significance level set at α = 0.05. No correction for multiple comparisons was applied. Therefore, all *p* values should be interpreted as exploratory, and findings with *p* < 0.05 were considered hypothesis-generating rather than confirmatory. Statistical analyses were performed using R (version 4.5.1; R Foundation for Statistical Computing, Vienna, Austria). 

## 3. Results

### 3.1. Patient Characteristics

A total of 272 MTP I arthrodeses in 252 patients were included, among which 149 were treated with screw fixation and 123 were treated with plate fixation. Patients treated with plate fixation were slightly older (60.4 ± 9.9 vs. 58.1 ± 9.1 years, *p* = 0.043), while other baseline variables were similar between groups (Table 1).

### 3.2. Radiographic Outcome Depending on Fixation Method

**Figure 2:**
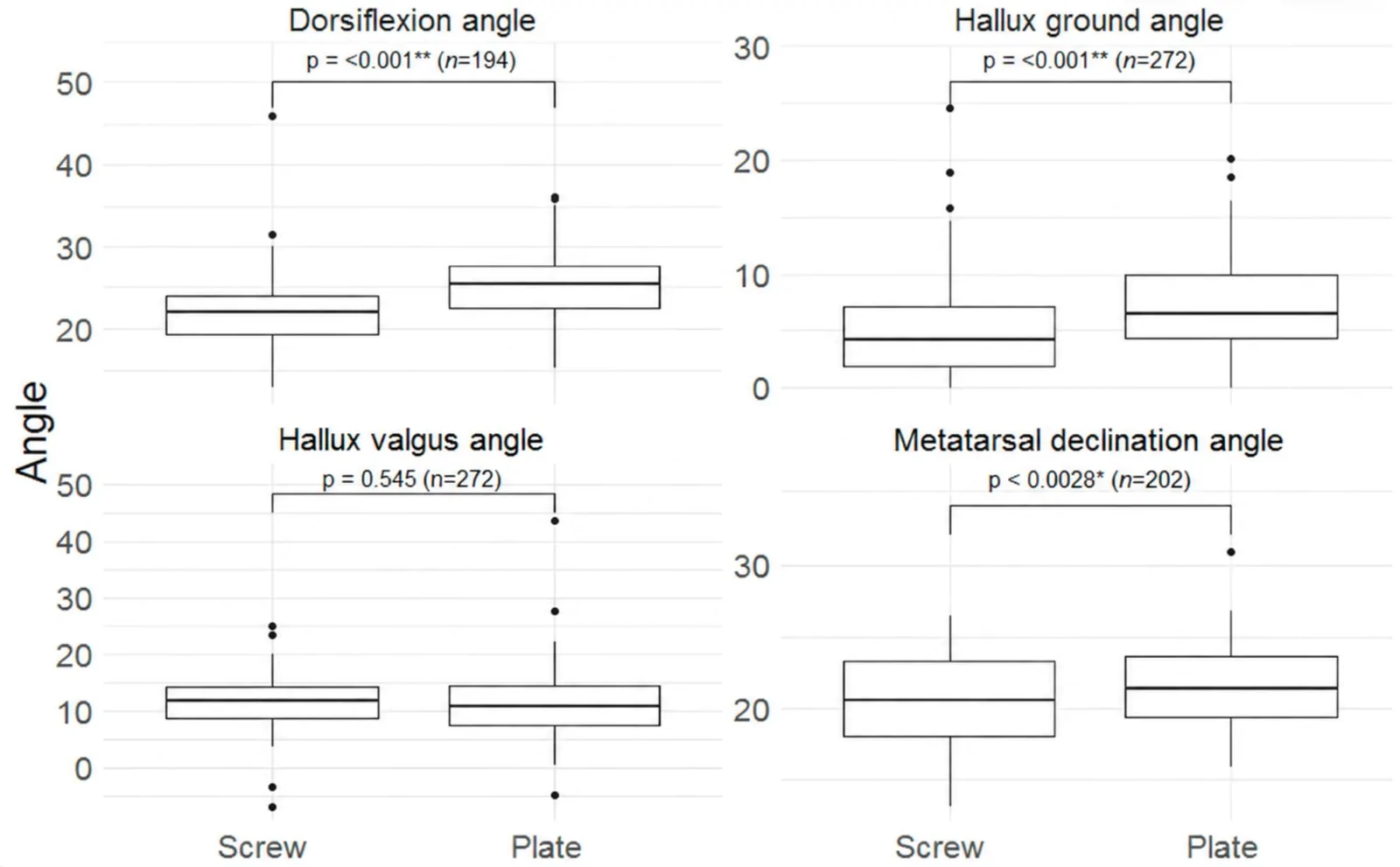
Boxplots of postoperative angular measurements by fixation method (screw vs. plate). *n* = sample size; * *p* < 0.05; ** *p* < 0.001.

Radiographic measurements were available for 272 arthrodeses for HVA and DFA, 202 forMDA, and 194 for HGA.The plate group demonstrated a higher metatarsal declination angle (MDA) than the screw group (21.3 degrees vs. 20.5 degrees; *p* = 0.028; 95% CI, 0.10 degrees to 1.90 degrees). Furthermore, the plate fixation group exhibited significantly greater dorsiflexion angle (DFA) (25.4 degrees vs. 21.9 degrees; *p* < 0.001; 95% CI, 2.40 degrees to 4.61 degrees) and hallux ground angle (HGA) (6.4 degrees vs. 4.15 degrees; *p* < 0.001; 95% CI, 1.10 degrees to 3.33 degrees). Hallux valgus angle (HVA) was comparable between groups (10.9 degrees vs. 11.95 degrees; *p* = 0.545; 95% CI, −1.0 degrees to 1.9 degrees) (Figure 2 and Table 2).

Given the higher metatarsal declination and postoperative DFA in the plate fixation group, a two-sided Pearson correlation analysis was performed and demonstrated a positive association between MDA and postoperative DFA in both fixation groups (screw: *r* = 0.190, *p* = 0.042; plate: *r* = 0.293, *p* = 0.006), indicating that greater metatarsal declination was associated with increased hallux dorsiflexion relative to the first metatarsal.

### 3.3. Surgeon-Level Analysis

Among surgeons, one was classified as high-volume (>30 procedures), four as medium-volume (10–30 procedures), and 18 as low-volume (<10 procedures). The high-volume surgeon was the department’s senior foot and ankle surgeon; the other groups included both consultants and residents. Surgeon case volume was associated with HGA (*p* = 0.002), with lower HGA values observed for the high-volume surgeon compared with medium- and low-volume surgeons (Table 3). DFA values were numerically higher in low- and medium-volume surgeons but did not differ between groups (*p* = 0.059). Native metatarsal declination angle did not differ statistically across surgeon case-volume groups (*p* = 0.954). Postoperative HVA did not differ statistically between groups (*p* = 0.148).When medium- and low-volume surgeons were pooled into a single group, the high-volume surgeon showed lower HGA (4.7 vs. 7.0 degrees, *p* < 0.001) and DFA values (22.5 vs. 23.8 degrees, *p* = 0.041), whereas MDA did not differ (*p* = 0.955).

### 3.4. Nonunion

Overall, 26 nonunions (9.6%) occurred. A progressive decrease in nonunion rates was observed with increasing surgeon volume (16.1%, 9.0%, and 6.6% for low-, medium-, and high-volume surgeons, respectively); however, this association did not reach statistical significance (*p* = 0.11). Nonunion rates were 7.4% after screw fixation and 12.2% after plate fixation, and did not differ significantly between groups (*p* = 0.18) (Table 4).

A surgeon-level analysis demonstrated considerable variability in surgeon-specific nonunion rates, particularly among low-volume surgeons; however, these estimates were based on small numbers of procedures per surgeon (Table 5). Overall, 57.7% of nonunions occurred in patients with at least one risk factor for impaired bone healing, most commonly nicotine use (42.3%).

## 4. Discussion

The present study suggests that postoperative sagittal alignment following first metatarsophalangeal joint arthrodesis is associated with multiple factors, including native anatomy, fixation method, and surgeon case volume.

While surgeon case volume was associated with sagittal alignment, the greater dorsiflexion observed in the plate group requires a more nuanced interpretation. Patients treated with plate fixation demonstrated a higher metatarsal declination angle, and a positive correlation between metatarsal declination angle and postoperative DFA was observed in both fixation groups. These findings suggest that the greater DFA observed in the plate group (25.4° vs. 21.9°, *p* < 0.001) was partly attributable to differences in native sagittal morphology rather than fixation method alone. Biomechanically, a more plantarflexed first metatarsal requires greater hallux dorsiflexion relative to the metatarsal to gain sufficient dorsiflexion for physiological rollover.

Native anatomy, however, does not fully explain the observed differences. In contrast to the DFA, the HGA is independent of first-ray declination, yet the plate group also demonstrated a more dorsiflexed hallux position relative to the ground (HGA 6.4 degrees vs. 4.15 degrees, *p* < 0.001). As arthrodesis position was established and compressed with an interfragmentary lag screw before application of the dorsal plate, which functioned as a neutralization construct, a direct mechanical effect of the plate on final alignment appears unlikely. Instead, the findings support an indirect influence of implant geometry on intraoperative positioning. Nearly two-thirds of the plate constructs used contoured dorsal plates. To accommodate the contour of these implants, surgeons may adjust the arthrodesis position before definitive fixation. Furthermore, temporary plate fixation with threaded K-wires draws the contoured plate onto the dorsal cortex and might subtly alter arthrodesis alignment if interfragmentary compression is not fully maintained. Previous studies have demonstrated that implant design and plate positioning can influence sagittal alignment following MTP I arthrodesis [20,21], with significantly higher dorsiflexion angles after plate fixation, as observed in our cohort [22]. Given that the dorsal cortical angle of the first metatarsal and proximal phalanx is smaller than the intramedullary angle, contoured dorsal plates predispose to greater hallux dorsiflexion [14], especially when the plate is placed proximal to its ideal position [20]. Collectively, these findings suggest that postoperative sagittal alignment may reflect the combined influence of native anatomy, implant geometry, and intraoperative positioning strategy.

The association between surgeon case volume and postoperative alignment further supports this concept. HGA differed significantly across surgeon case-volume groups, whereas DFA showed a numerical trend toward higher values in medium- and low-volume surgeons, reaching statistical significance when these two groups were pooled. Notably, native metatarsal declination did not differ across case-volume groups, suggesting that the observed differences in sagittal alignment were not attributable to differences in native sagittal anatomy. Instead, they may reflect differences in intraoperative assessment and positioning strategies. To our knowledge, this is the first study demonstrating an association between surgeon case volume and sagittal alignment following first MTP joint arthrodesis.

The clinical relevance of the observed radiographic differences should be interpreted with caution. In a previous study from our group in an overlapping cohort of 77 patients, neither DFA nor HGA correlated with AOFAS, FAOS or FFI scores after a mean follow-up of nine years [8]. Similarly, other authors reported no correlation between radiographic alignment and functional outcomes [9,23]. Chodaba et al. reported a correlation between dorsiflexion angle and improved outcomes in patients undergoing MTP fusion for hallux rigidus, but found no such association in patients undergoing MTP fusion for hallux valgus [24]. Moderate variations within the clinically accepted range therefore appear to be well tolerated, and the relevance of the present findings lies less in the small mean differences between groups than in the factors that drive alignment toward the margins of this range. Sagittal malalignment remains a major cause of dissatisfaction and reoperation [11], and excessive dorsiflexion has been associated with shoe conflict, impaired toe-off and increased metatarsal loading [12,14,15]. The present data identify three such factors that drive alignment toward these margins and can be addressed in planning and during surgery: native first-ray declination, which should be assessed on preoperative weight-bearing lateral radiographs and argues against a fixed DFA target; contoured plate geometry, which warrants re-assessment of hallux position after plate application; and limited surgeon experience. Notably, the mean HGA of the high-volume surgeon (4.7 degrees) closely matched the HGA corresponding to the recommended DFA range in our previous cohort (4.5 degrees) [8], whereas less experienced surgeons tended toward greater dorsiflexion. Although each factor shifted alignment by only a few degrees, their effects may add up, underlining the value of structured intraoperative assessment with simulated weight-bearing in surgical training.

The overall nonunion rate in the present study was 9.6%, which was comparable to previously reported rates [22,25,26,27,28]. Although nonunion occurred more frequently after plate fixation and among low-volume surgeons, neither association reached statistical significance, in line with a recent systematic review and meta-analysis reporting comparable union rates after screw and plate fixation [29]. However, considerable variability in nonunion rates was observed at the individual surgeon level, suggesting that surgeon-related factors contribute to fusion outcomes. These findings should be interpreted in the context of the evolving surgical practice during the study period. Initially, first MTP joint arthrodesis was performed almost exclusively by a single high-volume surgeon using compression screws. As the procedure became part of surgical training, plate fixation was increasingly adopted to provide additional construct stability for less experienced surgeons and eventually replaced screw fixation for all surgeons. Consequently, fixation method and surgeon case volume were closely linked, precluding a clear assessment of their independent effects on osseous union.

The present study has several limitations. Its retrospective design introduces potential selection bias and limits causal inference. The high-volume group consisted of a single surgeon, which may reflect individual technique rather than surgeon case volume alone. Intraoperative positioning was guided by simulated weight-bearing, rollover and the position of the toe tip. The latter, however, is influenced by interphalangeal joint motion, and the head of the proximal phalanx may represent a more reliable reference [30]. Functional outcome scores were not available for the present cohort; the clinical relevance can therefore only be inferred from prior work on an overlapping cohort [8]. Finally, radiographic measurements are subject to inherent variability and nonunion was assessed on plain radiographs only, without computed tomography or consideration of clinical symptoms.

Despite these limitations, the present study represents one of the larger clinical series evaluating sagittal alignment after first metatarsophalangeal joint arthrodesis and, to our knowledge, the first to investigate the combined influence of native anatomy, fixation method, and surgeon case volume. The findings suggest that anatomically contoured plates may facilitate fixation but should complement rather than dictate arthrodesis positioning. Likewise, radiographic target angles should serve only as reference values rather than fixed alignment goals, as the optimal sagittal alignment depends on the patient’s native anatomy. Hallux position should therefore be individualized according to native anatomy and confirmed by functional intraoperative assessment, including simulated weight-bearing.

## 5. Conclusions

Sagittal alignment following first metatarsophalangeal joint arthrodesis is influenced by native sagittal anatomy, fixation construct, and surgeon case volume. Rather than relying on implant geometry or predefined radiographic target values, hallux position should be individualized according to native anatomy and confirmed by functional intraoperative assessment.

## Figures and Tables

**Table 1:** Study population and baseline characteristics.

Variable	Overall	Screw	Plate	*p*	Test
*n*	272	149	123		
Age at surgery, years	59.1 (9.5)	58.1 (9.1)	60.4 (9.9)	0.043	*t*-test
Sex				0.663	Chi-square
Male	110 (40.4)	58 (38.9)	52 (42.3)		
Female	162 (59.6)	91 (61.1)	71 (57.8)		
Weight, kg	79.1 (14.7)	80.6 (15.0)	77.4 (14.1)	0.075	
Height, m	1.7 (0.1)	1.7 (0.1)	1.7 (0.1)	0.874	*t*-test
BMI, kg/m^2^	27.5 (4.6)	28.0 (4.6)	26.9 (4.5)	0.052	
Follow-up, months	39.8 (49.7)	40.7 (49.5)	38.5 (50.3)	0.720	
Side				0.676	Chi-square
Left	130 (47.8)	69 (46.3)	61 (49.6)		
Right	142 (52.2)	80 (53.7)	61 (50.4)		

Numbers are No. (%) unless otherwise noted. SD, standard deviation; BMI, body mass index.

**Table 2:** Radiographic parameters according to fixation method.

Parameter	Screw	Plate	95% CI for Difference	*p* Value
Hallux valgus angle, degrees	11.95	10.90	−1.0 to 1.9	0.545
Dorsiflexion angle, degrees	21.90	25.40	2.4 to 4.61	<0.001
Hallux ground angle, degrees	4.15	6.40	1.1 to 3.33	<0.001
Metatarsal declination angle, degrees	20.50	21.30	0.1 to 1.9	0.028

**Table 3:** Postoperative radiographic parameters according to surgeon case volume.

Parameter	Surgeon Experience	*n*	Mean	SD	*p* Value
HVA	>30 surgeries	121	12.18	4.10	0.15
	10–30 surgeries	99	10.86	6.70	
	<10 surgeries	52	12.21	5.28	
	Total	272	11.71	5.42	
DFA	>30 surgeries	121	22.49	3.56	0.06
	10–30 surgeries	99	23.91	4.80	
	<10 surgeries	52	23.64	6.09	
	Total	272	23.23	4.62	
HGA	>30 surgeries	87	4.73	3.32	0.002
	10–30 surgeries	73	7.12	4.50	
	<10 surgeries	34	6.77	6.00	
	Total	194	5.99	4.46	
MDA	>30 surgeries	90	20.89	3.05	0.95
	10–30 surgeries	77	21.03	3.04	
	<10 surgeries	35	20.93	2.89	
	Total	202	20.95	3.00	

**Table 4:** Nonunion rates according to surgeon case volume and fixation method.

	Surgeons (*n*)	Arthrodeses (*n*)	Nonunions (*n*)	Nonunion Rate (%)	*p* Value *
>30 surgeries	1	121	8	6.6%	0.11
10–30 surgeries	4	89	8	9.0%	
<10 surgeries	18	62	10	16.1%	
Total	23	272	26	9.6%	
Screw		149	11	7.4%	0.18
Plate		123	15	12.2%	
Total		272	26	9.6%	

* Chi-square test.

**Table 5:** Surgeon-level analysis of nonunion occurrence according to fixation method and patient risk factors.

Experience	Surgeon ID	Procedures	Screw	Plate	Nonunions, Total (Screw/Plate)	Nonunion Rate (%)	Risk Factor for Nonunion (%)
>30 surgeries	12	121	80	41	8 (5/3)	6.6%	62.5%
10–30 surgeries	22	30	0	30	5 (0/5)	16.7%	20%
	1	23	21	2	2 (1/1)	8.7%	100%
	23	23	0	23	1 (0/1)	4.3%	100%
<10 surgeries	21	7	7	0	1 (1/0)	14.3%	50%
	18	5	0	5	1 (0/1)	20.0%	100%
	7	3	2	1	1 (1/0)	33.3%	0%
	4	3	3	0	1 (1/0)	33.3%	0%
	6	3	1	2	1 (0/1)	33.3%	100%
	3	3	0	3	2 (0/2)	66.7%	100%
	9	2	2	0	1 (1/0)	50.0%	100%
	2	7	3	4	2 (1/1)	28.6%	0%

## Data Availability

The data presented in this study are available on request from the corresponding author due to privacy and ethical restrictions relating to patient data.

## Abbreviations

The following abbreviations are used in this manuscript:

MTP	Metatarsophalangeal
DFA	Dorsiflexion angle
HGA	Hallux ground angle
MDA	Metatarsal declination angle
HVA	Hallux valgus angle
AOFAS	American Orthopaedic Foot & Ankle Society
FAOS	Foot and Ankle Outcome Score
FFI	Foot Function Index
